# Small Cell Non-keratinizing Neuroendocrine Carcinoma Involving the Larynx and the Base of the Tongue: A Rare Case With a Locally Aggressive Spread

**DOI:** 10.7759/cureus.54272

**Published:** 2024-02-15

**Authors:** Nimisha Patil, Shraddha Jain, Smriti Wadhwa, Samarth Shukla, Preeti Mishra

**Affiliations:** 1 Department of Otorhinolaryngology - Head and Neck Surgery, Jawaharlal Nehru Medical College, Datta Meghe Institute of Higher Education and Research, Wardha, IND; 2 Department of Pathology, Jawaharlal Nehru Medical College, Datta Meghe Institute of Higher Education and Research, Wardha, IND

**Keywords:** laryngeal tumors, neuroendocrine carcinoma, tongue carcinoma, dysphagia, hoarse voice

## Abstract

Squamous cell carcinoma is the most predominant type of malignancy in the head and neck region with neuroendocrine carcinomas (NECs) being a rare occurrence. Here we report a rare case of small cell non-keratinizing NEC (WHO grade 3), TNM (tumor, node, and metastasis) stage T3N1M0, involving the larynx and the base of the tongue, in a 54-year-old male patient, demonstrating its rarity in an uncommon anatomical site and an aggressive and relatively uncommon pattern of spread for this tumor, over a period of two months. NECs in the head and neck region, especially those affecting the larynx and the posterior third of the tongue, remain exceedingly rare, comprising only a small fraction of malignancies in this region. The aggressive nature and distinct pattern of spread observed in this case underscore the importance of recognizing such unusual presentations for appropriate diagnosis and management. Given the rarity of this tumor type, a comprehensive understanding of its clinicopathological features is essential for guiding effective treatment strategies. We also discuss the treatment.

## Introduction

The most prevalent type of head and neck cancer is squamous cell carcinoma (SCC), forming almost ninety percent of all neoplasms, excluding non-melanoma skin cancer [1]. A rare form of malignancy in the head and neck is neuroendocrine carcinoma (NEC), representing only 0.3% of all head and neck cancers, and very few cases of it have been reported [2]. Squamous cell carcinoma represents over 80% of malignancies affecting the lip, tongue, tonsil, oropharynx, hypopharynx, larynx, and cervical esophagus, as well as 57% of nasal cancers. Conversely, nonsinonasal neuroendocrine carcinoma (NSNEC) occurrences in the head and neck region are extremely rare, with the larynx being the most commonly affected nonsinonasal site, albeit comprising only 0.5 to 1.0% of all laryngeal neoplasms [3]. However, its clinicopathological features remain incompletely characterized. The larynx is the most frequent site for NECs in the head and neck region and poses significant challenges due to their tendency to metastasize extensively, leading to a generally unfavorable prognosis [4]. Significantly, it is essential to note that the base of the tongue is recognized as a subsite of the oropharynx while the anterior two-thirds of the tongue have been defined as parts of the oral cavity [1]. Despite its histological similarity to conventional oral tongue carcinoma, a malignancy in the base of the tongue presents distinct implications that set it apart as a different disease entity concerning treatment, prognosis, and follow-up [1]. The posterior one-third of the tongue is a very rare site for the occurrence of NECs [5]. Here we report a case of small cell non-keratinizing type NEC of the larynx with the involvement of the base of the tongue, due to its rarity in this location and more aggressive course of presentation. 

## Case presentation

**Figure 1:**
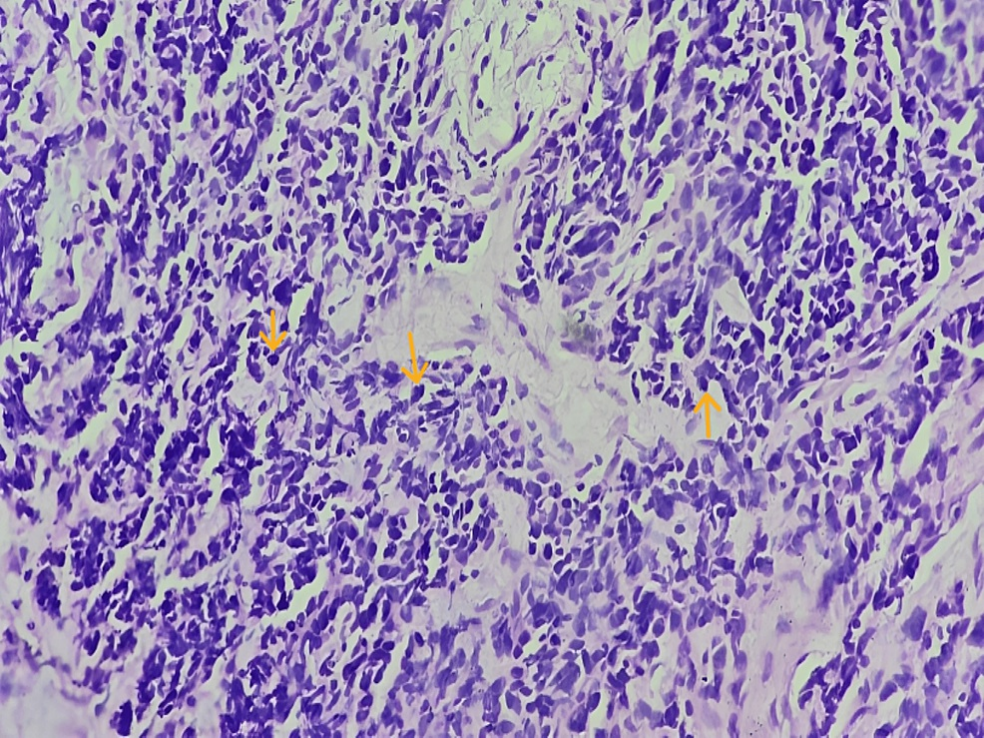
The photomicrograph of histopathological specimen at 40X view shows small-to-medium-sized, uniform cells with round-to-oval nuclei and finely granular chromatin. These cells are organized in trabeculae and some in rosette-like structures. Mitotic figures can be seen (shown by orange arrows)

**Figure 2:**
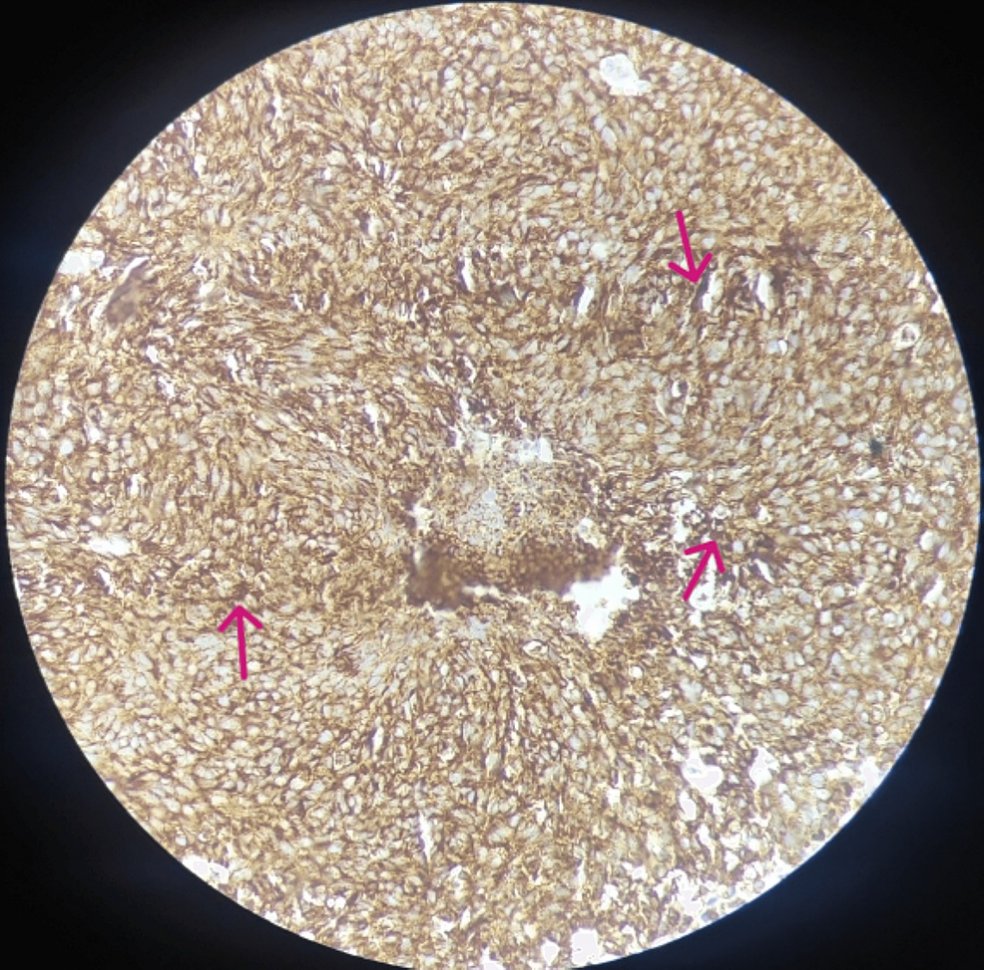
Immunohistochemical findings show tumor cells showing synaptophysin positivity (shown by red arrows)

We report a case of a 54-year-old man, who presented to the otorhinolaryngology out-patient department, with a two-month history of dysphagia and odynophagia for both solids and liquids associated with unremitting and gradually progressive change in voice leading to dysphonia grade 2 (moderate) as per the GRBAS (grade, roughness, breathiness, asthenia, and strain) scale over two months. The patient also complained of hyper-salivation, reduced appetite, and loss of weight for a one-month duration. There was no history of halitosis, nausea, vomiting, aspiration, or a burning sensation in the chest. His past history included tobacco consumption three to four times per day in the last 15 years and alcohol consumption for the last five to six years. On external examination, a 2 x 1.5 cm right level III cervical lymph node mass was palpable, which was hard in consistency with mild tenderness present. On a 70-degree rigid laryngoscopy, the gross features included an ulceroproliferative exophytic growth extending the right posterior aspect of the tongue, inferoposteriorly up to the right vallecula, pushing the epiglottis to the opposite side. The right false vocal cord, right pyriform fossa, and right aryepiglottic fold could not be visualized; the bilateral true vocal cords could not be visualized. Hence, the mobility of the cords could not be visualized. A biopsy was taken by a direct laryngoscopy under sedation and sent for a histopathology examination and immunohistochemistry. The gross macroscopic features on the histopathology included a nodular abnormality with a maximum anteroposterior diameter of 12 mm. It was diagnosed to be the deposits of a NEC (non-keratinizing small cell carcinoma) on a fine-needle aspiration cytology and histopathology (Figure 1) and an immunohistochemical study (Figure 2).

**Figure 3:**
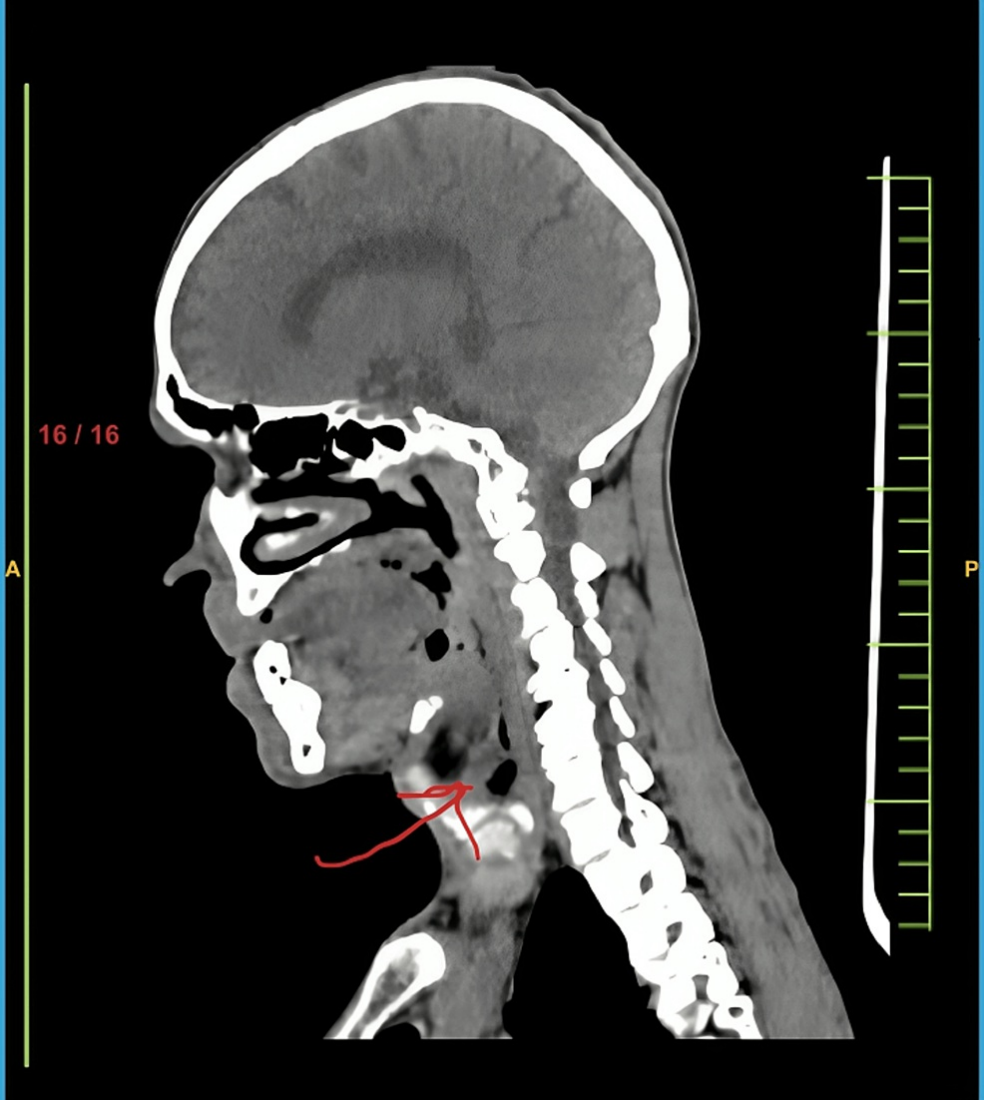
Contrast-enhanced CT scan showing a heterogeneously enhancing lesion (marked by a red arrow) in the supraglottic region

A contrast-enhanced CT scan showed heterogeneously enhancing soft tissue lesion density/thickening involving the posterior and the right lateral aspect of the tongue. The lesion was seen involving intrinsic muscles of the tongue, genioglossus, and hyoglossus of the right half of the tongue. The lesion was reaching up to the right tonsillar region, pharyngeal mucosal space, and parapharyngeal space with mild effacement of vallecula on both sides. There was a mild irregularity of the right lateral and anterior wall of the oropharynx with enhancement with the extension of thickening into the left pre-epiglottic space. The thickening of bilateral aryepiglottic folds was suggestive of the involvement of supra glottis (Figure 3).

**Figure 4:**
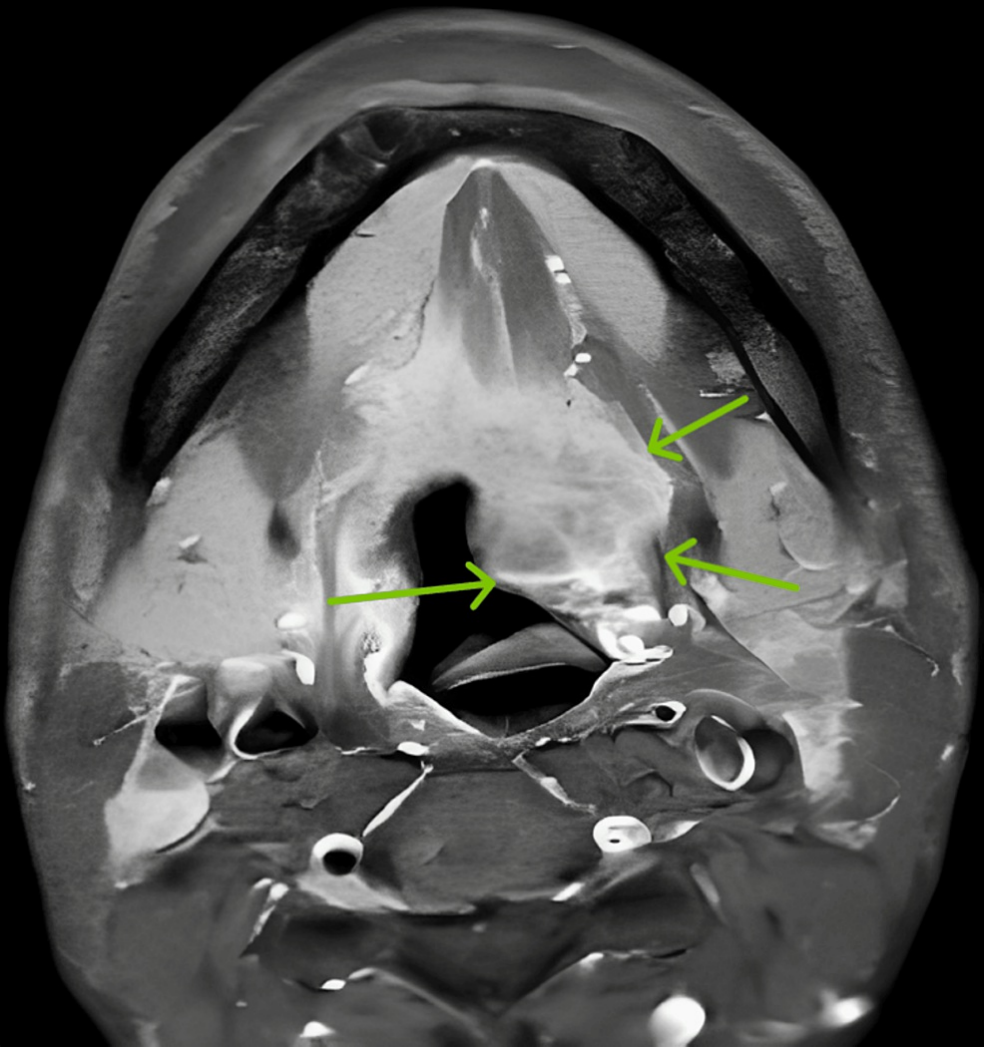
Contrast-enhanced MRI tongue with neck plain with contrast study shows the axial section of a post-contrast T1 fat saturation (FS) image showing a heterogeneously enhancing soft tissue intensity lesion involving bilateral genioglossus with a subtle post-contrast enhancement also seen in the supraglottic region (shown by green arrows)

A contrast-enhanced MRI (CEMRI) of the tongue was suggestive of a heterogeneously enhancing mass lesion in the tongue involving the posterior two-thirds of the tongue and of the bilaterally predominantly right inferolateral border appearing hypointense involving the floor of the mouth (Figure 4).

The patient exhibited metastases to the ipsilateral lymph nodes at the time of the diagnosis, but there was no distant metastasis to the lungs and liver, and the tumor at the base of the tongue involving the supraglottic region was diagnosed and staged as T3N1M0 as per TNM (tumor, node, and metastasis) staging. The patient received external beam radiation of 60 Gy in 30 fractions at 2 Gy per fraction over six weeks along with concurrent weekly cisplatin and etoposide chemotherapy six cycles. At six months of follow-up, the patient was doing well with a good response to treatment.

## Discussion

NECs are a group of rare and aggressive cancers that originate from neuroendocrine cells in various parts of the body [4]. These tumors can develop in a range of organs, including the lungs, gastrointestinal tract, pancreas, and other areas where neuroendocrine cells are present [5]. The nasal cavity, paranasal sinuses, nasopharynx, larynx, salivary glands, and middle ear are all affected by NECs of the head and neck, which are a group of heterogeneous epithelial neoplastic proliferations [6]. The supraglottis is a very often documented location for these carcinomas, while they may progress to other laryngeal regions as well. The larynx is the most common site for neuroendocrine carcinoma in the head and neck region, whereas cases in the posterior aspect of the tongue are rare [6]. In our case, the patient presented with dysphagia and hoarseness of voice initially, and later hypersalivation, so probably the tumor arose primarily in the supraglottis with a secondary involvement of the base of the tongue over a period of two months. This makes our case interesting, as NEcs of the larynx reported previously showed aggressive behavior with cartilage invasion and an extralaryngeal spread, or they were limited to the larynx [7]. The tumor in our patient showed an aggressive spread in a superior direction to involve a different subsite, that is, the base of the tongue, of the head and neck region over a period of two months. This behavior warrants attention, as it has treatment and prognostic implications.

According to the degree of tumor differentiation, NECs are classified as well-differentiated, moderately-differentiated, or poorly-differentiated entities in the WHO 2017 Classification of Head and Neck Tumors [8,9]. Poorly differentiated NECs are typically more aggressive and have a worse prognosis. The WHO 2017 Classification of NECs includes eight categories varying from well-differentiated NECs (Grade 1) and poorly-differentiated NECs, which fall under Grade 2 and are characterized by the presence of 10 or more mitoses per 10 per high-power fields (HPFs) or 2 mm², with a five-year disease-specific survival rate of 52.8%. Small cell type NEC is Grade 3 and has a five-year disease-specific survival rate of 19.3%. Large cell type NEC is Grade 3 and has a five-year disease-specific survival rate of 15.3% [8].

These tumors encompass different types that display diverse growth rates, ranging from slow-growing to rapidly proliferating. Our patient was diagnosed with a small cell non-keratinizing type of NEC (WHO Grade 3), TNM stage T3N1M0.

NECs are uncommon and have a variety of clinical demonstrations, making it difficult to diagnose and manage them. The diagnosis and treatment approach for NECs depend on the specific type, their location, hormone production, aggressiveness, and the presence of metastasis to other parts of the body [6]. 

Review studies on laryngeal NECs have indicated that surgical excision solely does not enhance local control and should be avoided even for early lesions. The clinical rule of thumb is that low-grade tumors are treated with surgical resection, whereas unresectable and symptomatic illness is treated with somatostatin analogues and/or interferon even though tumor regression with these medicines is uncommon [10]. For high-grade or metastatic NECs, etoposide/platinum-based chemotherapy remains the cornerstone of treatment; however, different drugs and methods are being investigated [10]. Hence it is very essential to detect early and start prompt treatment. 

According to published case studies, NECs of the tongue primarily affect middle-aged and elderly patients (aged 40-79 years), are more common in men, and are uncommon in women [11]. The mean age in NECs of the larynx is found to be around 70 years [9].

Malignant processes in the head and neck area are known for spreading locally into the regional lymph basin, particularly to sentinel lymph nodes, and lung metastasis is also common. Following the initial diagnosis, distant metastatic illnesses are usually found to have affected the lungs, bones, and brain [12,13]. In this case, there was a local spread to the bilateral submandibular and upper jugulodigastric region [10]. There was no evidence of distant metastasis in this case.

Our case presented with two primary subsites with local spread within two months, which made it challenging for us to plan the treatment. Radiotherapy alone has modest effectiveness in controlling tumors at the main location, but it does not enhance survival [2]. In our patient, there was involvement of the larynx and base of the tongue with local extension where it was difficult for surgical resection. We treated our patient with external beam radiation of 60 Gy in 30 fractions at 2 Gy per fraction over six weeks along with concurrent weekly cisplatin and etoposide chemotherapy of six cycles to which the patient responded well. The patient was followed up for six months and showed compliance with the above regimen.

## Conclusions

In conclusion, In our case, the patient presented with a hoarse voice and dysphagia, highlighting the clinical challenges associated with the diagnosis of an NEC in the head and neck. This case report contributes to the growing body of literature on the diverse presentations of NECs and emphasizes the need for further research to elucidate optimal therapeutic approaches. The management of such cases requires a multidisciplinary approach, considering the unique characteristics of NECs, and the need to tailor treatment plans to achieve the best possible outcomes. In summary, our report sheds light on the rarity and aggressive behavior of small cell non-keratinizing NECs in the larynx and base of the tongue, serving as a reminder for clinicians to remain vigilant in the face of atypical clinical presentations, ultimately contributing to improved diagnosis and patient care.
